# Active, Actuated, and Assistive: a Scoping Review of Exoskeletons for the Hands and Wrists

**DOI:** 10.33137/cpoj.v7i1.43827

**Published:** 2024-11-08

**Authors:** A. Galbert, A. Buis

**Affiliations:** Department of Biomedical Engineering, Faculty of Engineering, University of Strathclyde, Glasgow, Scotland.

**Keywords:** Upper Limbs, Exoskeletons, Assistive Devices, Wearable Devices, Design, Actuators, Outcome Measures, Systematic Review, Daily Activities, Wrist, Electromyography, Hand

## Abstract

**BACKGROUND:**

Assistive technology is often incorporated into rehabilitation and support for those impacted by upper limb impairments. When powered, these devices provide additional force to the joints of users with muscle weakness. Actuated devices allow dynamic movement compared to splints, therefore improving the ability to complete activities of daily living. However, these devices are not often prescribed and are underrepresented in research and clinical settings.

**OBJECTIVE:**

This review examined the existing literature on devices developed to support hand and wrist functionality in daily activities. Focusing on active, powered, and actuated devices, to gain a clearer understanding of the current limitations in their design and prescription.

**METHODOLOGY:**

The scoping review was conducted using the PRISMA-ScR guidelines. A systematic search was done on MEDLINE, EMBASE, Scopus, Web of Science, and NHS the Knowledge Network from inception to May 2023. Articles were included if the device was portable; supported the hands and wrist actively using an actuator; and could be used for assistive living during or post-rehabilitation period.

**FINDINGS:**

A total of 135 studies were included in the analysis of which 34 were clinical trials. The design and control methods of 121 devices were analyzed. Electrical stimulation and direct mechanical transmission were popular actuation methods. Electromyography (EMG) and joint movement detection were highly used control methods to translate user intentions to device actuation. A total of 226 validation methods were reported, of which 44% were clinically validated. Studies were often not conducted in operational environments with 69% at technology readiness levels ≤ 6, indicating that further development and testing is required.

**CONCLUSION:**

The existing literature on hand and wrist exoskeletons presents large variations in validation methods and technical requirements for user-specific characteristics. This suggests a need for well-defined testing protocols and refined reporting of device designs. This would improve the significance of clinical outcomes and new assistive technology.

## INTRODUCTION

Upper limb impairment, resulting from a range of factors such as injury, neurological disorders, diseases, conditions, and general comorbidities, can have a profound and detrimental impact on an individual's overall quality of life.^1^ This impairment often leads to significant limitations in physical activity^2,3^ and can contribute to mental health challenges, due to the loss of independence and functionality.^4^ Symptoms such as muscle weakness, reduced muscle control, neurological issues, and prehension difficulties vary in severity and permanence.

Due to this variability, a one-size-fits-all approach is inadequate. Tailored rehabilitation programs and assistive interventions must be designed to accommodate the specific requirements of individuals, enabling them to perform activities of daily living (ADLs) more effectively and improving their overall well-being.

Spasticity, muscle weakness and prehension difficulties affect the upper limb differently. Spasticity is defined as velocity-dependent resistance,^5^ due to this muscle contracture, impaired control of voluntary hand-opening tasks and activities is seen.^4^ In contrast, muscle weakness affects hand-closing tasks such as grasping utensils and opening doors. The hands are the only prehensile organ in the human body.^6^ Prehension is required for feedback during tasks and coordination, therefore reduced prehension disrupts the balance between power and precision requirements of dexterous tasks.^7^ When a person receives no feedback during functional tasks, they may be unable to gauge if they have optimal hand orientation or enough strength to hold an item. Hand and wrist impairments of all types target a person’s ability to perform ADLs.

In addition to performing ADLs, biopsychosocial factors are also impacted by hand impairment.^8^ The biopsychosocial model is a concept which allows for the classification of factors which may contribute to any individual’s mental and physical health.^9–11^ The psychological impact of hand impairment can be presented as distress, depression, and low self-efficacy. Persons with hand impairments have also shown a reduction of measures determining quality of life.^2^ Sense of freedom, belonging and security are major social factors affected by having upper limb impairment.^8^ These people may also have reduced independence and may rely on family, caregivers, and allied health professionals for support. The biopsychosocial factors mentioned introduce a global burden on resources, cost, time and availability of support.^12–14^ Fortunately, assistive technology may reduce that burden while also attaining sustainable development goals for the future ageing population affected by these impairments.^15,16^

To facilitate upper limb functional tasks, interventions such as rehabilitation and assistive technology may be provided. The objective of assistive technology is to ensure safety, and accessibility, promote independence and improve quality of life. To achieve these objectives, devices must be tailored to the user’s requirements. For users who require augmented strength and functionality to perform tasks, a powered and actuated device would be appropriate. Examples of active devices include exoskeletons and exosuits.^17^ The introduction of actuators makes the device active, compared to passive devices that use elastics, levers and springs to support user motion such as dynamic orthosis. These devices function by applying force from an actuator on segments of the upper limb. Actuators are devices which convert energy to motion; this energy may be electric such as DC motors. Depending on the position and power of the force applied to the upper limb, the device can assist in various functional tasks.

The evolution of upper limb assistive devices has had rapid advancements in technology. It has grown in popularity within the commercial sector as workplace health and safety systems, and as stationary end-effector devices within physical rehabilitation settings.^18^ Despite the advantages of using these devices,^16,19,20^ the National Service Framework for Long-Term Conditions and Clinical Commissioning Groups (national to the United Kingdom) have minimal to absent policies for using these motorized devices.^21^ The rationale behind this regulatory stance is uncertain. However, global reports on assistive technology have postulated several factors for the general lack of prescription of assistive devices including limited-service provision, inadequate products, market shortcomings, governance and funding constraints, as well as sociodemographic barriers.^16^ These factors may apply to actuated devices, but these reports^16,21^ do not focus on actuated devices.

Furthermore, literature reviewing the upper limb exoskeletons rarely discusses the hands and wrist segments,^18^ and of those which have, there is a lack of breadth on clinical utility and outcome measures.^17,20,22,23^ Based on the gaps in global reports and review literature, a study summarizing actuated devices would be appropriate.

This scoping review aimed to explore the research question: What is known about active actuated and assistive devices for the hands and wrist? The secondary objectives include: **1**) Defining the intended populations of these devices, **2**) Abstracting an overview of the device design: including modes of actuation, user intention methods and force transmission methods, **3**) Summarize and categorize validation strategies used in the study of these devices.

## METHODOLOGY

A scoping review summarizing the breadth of existing literature concerning active, actuated (powered and motorized) and assistive (provides support during functional tasks) devices designed for the hands and wrists was conducted. The scoping review offers a methodological approach to survey the evidence, key concepts, and analyze knowledge gaps.^24–27^ This may illuminate potential rationales for the underrepresentation of hand and wrist assistive devices in literature. It may also ascertain if the barriers outlined in the global report on assistive technology^16^ apply to actuated devices.

A scoping review was chosen as it maps out the extent of existing research on a broad topic. For this study, it is active assistive devices for hand and wrist actuation. Scoping reviews have more inclusive eligibility criteria compared to systematic reviews. This encourages the use of larger sources of literature, more time-effective analysis, and provides evidence for future systematic reviews.

This scoping review follows the Preferred Reporting Items for Systematic Reviews and meta-analysis extension for Scoping Reviews (PRISMA-ScR).^28^ This extension is an update from the PRISMA guidelines which is a validated systematic approach for evidence syntheses.^24^ The search criteria for the database were structured according to Population, Concept, Context (PCC) framework.^29^ The population was defined as individuals experiencing hand and/or wrist impairment. The concepts focused on devices with active actuation and power. The context encompassed devices which assist ADLs during and post-rehabilitation. The definition of post-rehabilitation in this study refers to the phase of recovery and support that follows an initial rehabilitation program.

### Database Search

Five databases were searched from inception to the date of search (May 25th, 2023). The databases selected were MEDLINE (Ovid), EMBASE (Ovid), Scopus, Web of Science, and NHS the Knowledge Network. No limitations or filters were applied to the results during the systematic database search. These databases were chosen based on their optimal combination, and collectively satisfy the minimum requirement of databases necessary to ensure adequate and efficient coverage of studies.^30,31^ Search terms were combined with Boolean logic ((Hand OR hands OR extremity) AND (Wrist OR wrists OR carpus) AND (Device OR devices OR assistive devices OR actuated devices OR powered devices OR exoskeleton OR glove OR dynamic) AND (Functional OR function OR assist OR assistive OR assistance OR aid OR aiding OR support)).

Database search results were imported to Endnote v20 in an RIS file format. Duplicates and retractions were removed using Endnote v20 software.

### Selection Criteria

Two screening processes were used: The first examined titles and abstracts for all papers on Microsoft Excel 2018 Version 2409. The inclusion criteria were “Is this an active, actuated, and assistive device for the hands and wrist?”. Papers were marked “include”, “exclude”, “duplicate” and “maybe”. The process of tagging studies was conducted by 2 reviewers with 86.9% agreement, and any disagreements were resolved with consensus. All studies tagged as duplicates were checked to ensure a version was kept within the dataset. The second screening process examined the full paper against the inclusion criteria shown in **Table 1**. The studies were tagged with include or exclude using these criteria.

**Table 1:** Exclusions Criteria for Second Screening.

Decision Tag	Exclusion Criteria	Additional Notes
**Reason 1:**	Is the device mobile?	Devices grounded to static tables are excluded, but devices mounted to wheelchairs are included as it is mobile.
**Reason 2:**	Does the device actively support hand and/or wrist movement?	Devices which immobilize joints are excluded. Devices which support the wrist in a static position and do not support hand movement are also excluded.
**Reason 3:**	Is this a complete system?	A complete system must include hardware and software.
**Reason 4:**	Does the device support ADLs?	If the hand and wrist are put in a static position, it can be assumed ADLs are not being completed and therefore excluded. Devices which train the hand/wrist for ADLs are included.
**Reason 5:**	Is the study primary research and not a review?	Excludes all reviews; examples include systematic, scoping, narrative, and state-of-the-art reviews.
**Miscellaneous:**	Access to full paper in English	Excludes research posters, published abstracts, and conference abstracts. Excludes papers not provided with English translation.

### Data Extraction

A total of 24 data items were charted independently by researcher AG. The full list of data charting items collected, and their definitions can be found in **Table 2**. Records from the same research group were considered individually if the devices described were mechanically different from each other, whereas articles regarding different iterations of the same device were grouped with the latest prototype iteration considered. For records using the same device, the most representative across all papers was chosen.

**Table 2:** List of all data items collected, and their definitions.

Data Item	Definition
**Title**	Title of the article as found in the database.
**Reference ID**	Reference number linked to list of all referenced in the dataset.
**Author**	List of all authors.
**Year**	The year the article was published.
**Country of Study**	The country of study is either given based on the institution or location of the clinic of the affiliated author.
**Study Type**	The study type was defined by the publisher. Options cited include articles, research papers, case reports, letters, and pilot studies.
**Method**	Methodology of the study.
**Sum of Participants**	The sum of the participants in the study.
**Male**	The sum of male participants (when provided).
**Female**	The sum of female participants (when provided).
**Age Range**	Based on the participants, the youngest to oldest participants make the age range.
**Target Population**	The intended population/user group for the device.
**Grouped Target Population**	To reduce variations in a target population, the grouped target was separated into 22 subgroups with 13 unique groups that were often combined: Autoimmune Disease Cardiovascular Disease (CVD)/Stroke Entrapment Neuropathy Healthy Joint Disorder Muscular Dystrophy Musculoskeletal Impairment Musculoskeletal Injury Neurological Disorder Sarcopenia Spinal Cord Injury (SCI) Traumatic Brain Injury (TBI) Tremor
**Study Population**	The condition of the participants in the study.
**Device**	Name of the device if provided.
**Weight of Device (g)**	Weight of the device on the upper limb unless specified otherwise.
**DoF (Degree of Freedom)**	DoF of the entire device refers to the number of independent ways the mechanical transmission can move joints in the hand/wrist.
**Mechanical Transmission**	Method of applying active force from an actuator to the joint of the user.
**Grouped Mechanical Transmission**	To reduce variations in mechanical transmissions, sub-classes were Grouped into 6: **Cable-Conduit:** These systems use cables or flexile wires inside a conduit. They transmit force in push or pull motions, like a brake cable on a bicycle. **Direct:** These systems transfer force directly to the joint segment, for instance, a linear actuator may push the wrist into flexion. **Fluidic Transmission:** Commonly hydraulic or pneumatic, these systems use pressurized fluids in a tube to control movement. **Muscle Contraction:** To induce movement, electrical stimulation is used to contract (shorten) the muscle. The placement of electrical stimulation triggers various joint movements. **Pulley:** As a motor turns, the pulley system amplifies the force and moves the joint segment attached to the system. **Supernumerary:** These systems include extra robotic limbs such as fingers or hands. These devices aid in functional tasks by providing additional force.
**Hand/Wrist**	Is the device aimed to support the hand, the wrist, or the hand and wrist together?
**User Intent/Detection Methods**	The user intent/detection methods are how the device is controlled. The user will actively trigger the device, this can be by using a joystick, by contracting muscles, and many more.
**Outcome Measures**	All outcome measures and outcome measurement tools that were used in the study.
**Technology Readiness Level (TRL)**	The TRL was assigned according to the TRL definition provided by the HORIZON 2020 - Work Program 2014–2015 defined in **Table 4**. The TRL is a scale used to measure how developed and ready a technology is for practical use.
**Outcome Measure Field**	The outcome measures were separated into clinical, technical, or clinical and technical measures. Clinical outcomes focus on the patient’s health and quality of life, technical outcomes focus on the functionality and performance of the device. Classification of outcome measures was aided by the WHO ICF Model.^11^

The data charting items provided a comprehensive summary of participants demographic features, interventions, validations, and technology readiness levels (TRLs) of the included studies. Participants demographics include country, the sum of participants, gender, age, and patient conditions (if applicable). The intervention comprises device name, weight, degree of freedom (DoF), mechanical transmission, user intent/detection methods, and limb segment the device supports. The synthesis of validation includes both clinical outcome measures and non-clinical. TRLs were also part of the data extraction and can be analyzed against all data items to investigate potential trends in technological advancements.

## RESULTS

### Overview

**Figure 1:**
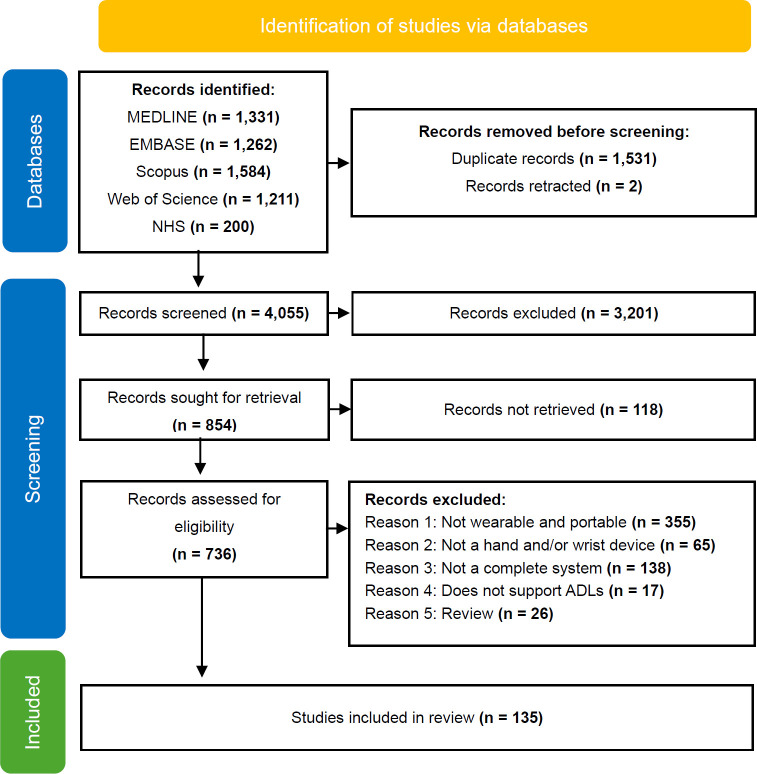
PRISMA Flowchart of database search, inspired by PRISMA2020.^39^

A total of 5,588 records were identified from the initial database search conducted in May 2023, of which 135 studies were included in the scoping review dataset.^32^ The selected studies were published between 1995 to 2023 (m = 2016, SD = 6.64), with 54% (73/135) studies published in the last 5 years. The selection process is provided in **Figure 1**. Two publications were identified and retracted using EndNote software.

The most popular methodology used an experimental design (25%, 34/135), followed by feasibility studies (21%, 28/135). Clinical methodologies, such as RCTs (Randomized Control Trials, n = 12) and single group trials (n = 12), made up 34% (46/135) of the dataset.

Thirty-one countries contributed to the field of hand and wrist exoskeletons. Of which, the USA (20%, 27/135), China (16%, 21/135), Japan (12%, 16/135), Italy (8%, 11/135) and South Korea (6%, 8/135) produced the highest number of studies.

Following the World Development Indicators for income classification,^33^ 3 studies were completed in Low-Middle Income Economies,^34–36^ 31 in Upper-Middle Income and 101 in High Income. The correlation (r) between the number of studies published per country and the sum of participants was foreseeably high (r=0.867). An outlier to this trend is one study from Russia by Abramovich et al,^37^ which included 96 participants. This was also the second largest sum of participants in one study, with the largest sum of participants in a study conducted by Takebayashi et al with 115 participants.^38^

### Participants

The sum of participants within the dataset totaled 1310. Of the 1310 participants (female: male 39%:61%), 46% (597/1310) had upper limb impairment due to stroke, 28% (371/1310) have been affected by Spinal Cord Injury (SCI) in the form of tetraplegia, hemiparesis, or hemiplegia, and 11% (140/1310) were considered healthy. The least reported conditions for support included persons with Cerebral palsy^40^ with 19 participants, upper limb tremors^34,41^ with 20 participants, Parkinson's disease^36^ with 10 participants, and support post-burns^42^ with 20 participants.

Of the 39 studies which recruited healthy participants solely, two devices^43,44^ were intended for human augmentation in healthy user groups. Age of participants ranged from 12–83 years old: two studies^45,46^ included a device for non-adults.

### Intervention

In all, 121 devices were presented within the studies. A summary of the devices is presented in **Table 3**. Devices were categorized by their Weight (g), Degree of Freedom (DoF), Power Transmission, Mechanical Transmission, segment of support (Hand and or Wrist) and User intent. Of the target support joint, 37% (45/121) of devices supported hand actuation, 36% (44/121) supported both the hand and wrist and 26% (32/121) supported wrist actuation only.

**Table 3:** Summary of devices analyzed.

Device name	Reference	Weight of Device on Arm (g)	DoF	Power Transmission	Mechanical Transmission	Hand/Wrist	User Intent
2-channel portable battery47operated FES system	47	–	3	Electrical stimulation	Muscle contraction	Hand and wrist	EMG signal
3-CRP	48	2700	3	DC motors	Direct	Hand and wrist	Concurrent movement
4-DOF wheelchair exoskeleton and Carbon hand	49	4000	4	Maxon DC motor	Cable and gear	Hand and wrist	Joint position and tactile
A5 hand function training system	42	–	6	Linear actuator	Bar linkage	Hand and wrist	Muscle torque
Anthropomimetic upper limb assistive device	35	–	12	DC motors	Pulley	Hand and wrist	Manual selection
Armeo Power II	50	205000^*^	7	Motors	Gears	Wrist	Joint torque
Attention-controlled wrist rehabilitation method	51	415	2	Linear actuator	Push-pull cable	Wrist	EEG signal
BOTAS	52	–	6	Electrical stimulation	Direct	Hand and wrist	EMG signal and EEG signal
BRIDGE EMPATIA	53	–	5	Stepper motor	Bar linkage	Wrist	Manual selection (joystick)
DiaDENS-PKM	54	350	–	Electrical stimulation	Muscle contraction	Wrist	EMG signal
Distributed FES and Assessment System	55	–	2	Electrical stimulation	Muscle contraction	Hand and wrist	Concurrent EMG signal and finger angle
DTF Splint	56	–	1	Pneumatic actuator	Pneumatic	Hand	Manual selection
DTSaM Orthosis	57	–	2	Pneumatic actuator	Pneumatic	Wrist	Joint angle
DULEX-II	58	504	3	pneumatic and linear actuator	Pneumatic	Hand and wrist	concurrent EMG
Electrical stimulation	59	–	–	Electrical stimulation	Muscle contraction	Wrist	Manual selection
Electromechanical orthosis and MyoSystem BrI system	60	–	2	DC motors	Pulley	Hand and wrist	EMG
EMG-driven exoneuromusculoskeleton	61	368	–	Pneumatic actuator	Pneumatic	Hand	Muscle torque
EMG-driven NMES-robotic arm	62	–	–	DC servo motors	Direct	Wrist	EMG signal
EMG-Driven NMES-Robotic Hand	63	–	4	Linear actuator	Bar linkage	Hand	EMG
EMG-driven WH-ENMS	64	–	5	Pneumatic actuator	Pneumatic	Hand and wrist	EMG
Emotiv EPOC and Rehastim	65	–	–	Electrical stimulation	Muscle contraction	Hand and wrist	EEG signal
Empi FOCUS	66	–	–	Electrical stimulation	Muscle contraction	Hand and wrist	Manual selection
EMS 400 and Ultraflex	40	–	2	Eletrical stimulation	Muscle contraction	Wrist	Manual selection
Energy-efficient wrist exoskeleton	67	–	1	Pneumatic actuator	Pneumatic	Wrist	Joint angle
ETS-MARSE	68	7072	7	Brushless DC motors	Gears	Wrist	Muscle torque
eWrist	69	556	1	Brushless DC motors	Gears	Wrist	Joint angle and EMG signal
ExoFinger	70	–	2	DC servo motors	Bar linkage	Hand	EMG signal, Finger temperature and Joint angle
EXOTIC upper limb exoskeleton and ITCI and Carbon hand	71	6000	4	Maxon DC motor	Cable and gear	Hand and wrist	Manual tongue
Exo-Wrist	72	1003	2	Rotary encoder	Pulley	Wrist	Muscle torque
EXTEND exoskeleton	73	105	3	Linear actuator	Bowden cable	Hand	Manual selection
Fesia grasp Device	74	91	8	Electrical stimulation	Muscle contraction	Hand and wrist	EMG signal
FESMATE CE1230	75	–	–	Electrical stimulation	Muscle contraction	Hand and wrist	EMG
FESMED 4050 device	76	200	–	Electrical stimulation	Muscle contraction	Hand and wrist	Manual selection
Five-digit 3D printed battery-powered and force augmenting orthotic exoskeleton	77	–	–	Linear actuator	Cable	Hand	Muscle torque
Five-fingered exoskeleton hand	78,79	2000	3	DC motors	Bar linkage	Hand and wrist	EMG and wrist joint angle
Flexohand	80	280	6	DC servo motors	Bowden cable	Hand	Manual selection
Foot-controlled hand/forearm exoskeleton	81	–	4	DC servo motors	Pulley	Hand and wrist	Manual Foot selection
GBBAs	82	95	3	Pneumatic actuator	Pneumatic	Hand	Joint angle and muscle torque
Gloreha lite glove	83	80	5	Pneumatic actuator	Pneumatic	Hand	Manualselection
Glove-based assistive device	84	–	2	Pneumatic actuator	Pneumatic	Wrist	Wrist movement
GraspyGlove	85	340	4	Maxon DC motor	Push-pull cable	Hand	Sensor proximity
Hand assistive device	86	–	1	Linear actuator	Bowden cable	Hand	Muscle torque (index)
Hand exoskeleton	87	114	3	DC motors	Bowden cable	Hand	Joint angle and muscle torque
Hand exoskeleton system HES	88	350	2	DC servo motors	Bar linkage	Hand	Manual hand
Hand function rehabilitation robot	89	450	2	Linear actuator	Bar linkage	Hand	Manual hand (touch screen)
Hand/Wrist exoskeleton	90	1815	7	DC Torque motor	Bar linkage	Hand	EMG and joint motion
HANDS therapy	91,92	–	–	Electrical stimulation	Muscle contraction	Hand and wrist	EMG signal
Hybrid system	93	402	5	Linear actuator	Bar linkage	Hand	EMG signal and EEG signal
Hybrid-driven compliant hand exoskeleton	94	147	–	DC Torque motor	Cable	Hand	Finger torque
Implanted sensor-controlled microstimulator system	95	–	–	Electrical stimulation	Muscle contraction	Hand and wrist	EMG signal
INTFES	96	170	–	Electrical stimulation	Muscle contraction	Hand and wrist	EMG signal
intracortical MEA-BCI-FES	97	–	–	Electrical stimulation	Muscle contraction	Hand and wrist	EEG signal (Implant)
IOTA	98	230	2	DC servo motors	Cable	Hand	Manual hand
Layer jamming-based soft Tremor Suppression Glove	34	30	6	DC servo motors	Hydraulic	Hand	Tremor
MAH system	99	580	6	DC servo motors	Supernumerary	Hand	Wrist angle
MAHI Exo-II	100,101	340	4	DC motors	Bar linkage	Wrist	Manual selection
MeCFES	102	–	2	Electrical stimulation	Muscle contraction	Hand	EMG wrist
MeFES	103	–	–	Electrical stimulation	Muscle contraction	Hand and wrist	EMG signal
Mirror hand HS 001	104	800	5	Motors	Bar linkage	Hand	Mirrored motion
Mirror-image motion device with an exoskeleton	105	1800	3	Brushless DC motors	Cable	Wrist	Mirroredmotion
Motor orthotic device	106	–	1	Ultrasonic motor	Gears	Wrist	EMG signal
MWDO	107	330	2	DC motors	Bar linkage	Hand and wrist	Wrist torque
Myoelectric control	108	–	2	Electrical stimulation	Muscle contraction	Wrist	EMG
MyoPro	109–112	1814	2	Motors	Direct	Hand and wrist	EMG signal
NESM and 5-DOF wrist-hand exoskeleton	113	–	9	DC motors	Bar linkage	Hand and wrist	Joint position
NESS handmaster system	114–116	–	–	Electrical stimulation	Muscle contraction	Hand	Manualselection
Neuro-orthosis	117,118	–	2	Electrical stimulation	Muscle contraction	Wrist	Joint angle
NMES-robot arm	119	895	2	DC Torque motor	Muscle contraction and direct	Wrist	EMG signal and NMES signal
Odstock 2-channel Programmable Stimulator	120	200	–	Electrical stimulation	Muscle contraction	Hand and wrist	EMG signal
Paediatric hand exoskeleton PEXO	45	107	1	Linear actuator	Cable	Hand and wrist	Manual hand OR hands-free voice control based on keyword detection
Pinch assistant	121	580	5	DC servo motors	Pulley	Hand	Index andthumb torque
Pinotti portable robotic exoskeleton PPRE	122	1600	2	DC motors	Gears	Hand and wrist	Manual hand
PneuGlove	123	–	2	Pneumatic actuator	Pneumatic	Hand	Joint angle
Pneumatic-controlled finger extension system	43	2000^*^	1	Pneumatic actuator	Pneumatic	Hand	EEG signal
Power augmentation soft glove	124	120	4	Pneumatic actuator	McKibben	Joint torque (index)
Power-assisted FES	125	–	3	Electrical stimulation	Muscle contraction	Hand and wrist	EMG signal
REHA 2030	126	–	1	DC motors	Bar linkage	Wrist	Wrist angle and velocity
ReIn-hand system (Empi 300 and EMG collection unit)	127,128	227	–	Electrical stimulation	Muscle contraction	Hand and wrist	EMG signal
RELab tenoexo	129,130	148	3	Maxon DC motor	Bowden cable	Hand	Finger torqueand bend
ReoGo-J	38	79000^*^	3	Motors	Direct	Wrist	Manual selection
Rope-driven flexible robot	131	–	–	Linear actuator	Pulley	Hand	Manual selection (touch screen)
RUPERT IV	132,133	–	5	Pneumatic actuator	Pneumatic	Wrist	Joint positionand tactile
SaeboFlex and BMR Neurotech electrical stimulator unit	134	1587	5	Electrical stimulation	Muscle contraction	Hand and wrist	Muscle torque
SaeboMAS and accelerometer-triggered FES	135	–	–	Electrical stimulation	Muscle contraction	Hand and wrist	Joint position
SCRIPT Active orthosis SAO-i3	136	–	3	DC motors	Bar linkage	Hand and wrist	Joint angle
SCRIPT1 Project	137	–	–	Elastic torque	Pulley	Hand and wrist	Wrist motion and muscle torque
SEM Glove	138	700	3	Brushless DC motors	Bowden cable	Hand	Fingertip tactile
Semi-soft assistive glove SAG	139	–	2	DC motors	Cable	Hand	Wrist motion and EMG
SETS system	41	255	3	Flexible semiactive actuator	Direct	Wrist	Tremor
SMA muscle	140	300	2	SMA	Hydraulic	Wrist	Manual selection
SNU Exo-glove	141	–	3	Brushless DC motors	Cable	Hand	Joint velocityand joint tensile
Soft glove	142	237	6	Pneumatic actuator	Pneumatic	Hand andwrist	Manual selection
Soft modular elbow-wrist rehabilitation exoskeleton driven by PAMs	143	–	2	Pneumatic actuator	Pneumatic	Wrist	Joint position
Soft robotic rehabilitation glove	144	–	–	Pneumatic actuator	Pneumatic	Hand	Manualselection
Soft sixth finger	145,146	140	1	DC servo motors	Supernumerary	Hand	EMG
SoftHand X system	147	500	–	Maxon DC motor	Supernumerary	Wrist	Joint angle (finger)
SR Fingers	148	750	6	DC servo motors	Supernumerary	Hand	Hand position
SSVEP-BCI controlled soft robotic glove rehabilitation system	149	–	2	Pneumatic actuator	Pneumatic	Hand	EEG signal
Super stim ZZAEV906	46	–	3	Electrical stimulation	Muscle contraction	Hand and wrist	EMG signal
Supernumerary robotic finger SRF	44	650	6	DC servo motors	Supernumerary	Hand	Joint angle
TCAMs-Exo	150	135	2	DC motors	Artificial muscle	Wrist	EMG and wrist joint angle
tDCS	151	–	–	Electrical stimulation	Muscle contraction	Hand and wrist	EMG signal
TDS-HM the hand mentor and tongue drive system	152	–	2	Pneumatic actuator	Pneumatic	Hand andwrist	Tongue position
TENS Stimulator N604	153	–	–	Electrical stimulation	Muscle contraction	Hand and wrist	EMG
T-GRIP exoskeleton	154	50	1	Linear actuator	Bar linkage	Hand	Joint angle (wrist)
The Bionic glove	155	–	–	Electrical stimulation	Muscle contraction	Hand	Wrist position
The Hand exoskeleton	156	1800	15	Linear actuator	Push-pull cable	Hand	Mirrored motion
TIGER	157,158	420	2	Brushless DC motors	Bar linkage	Hand andwrist	Manual hand (touch screen)
Upper limb rehabilitation robot	159	–	6	DC Motors	Gears	Wrist	Manual selection
Utah microelectrode array and NMES	160	–	6	Electrical stimulation	Muscle contraction	Hand and wrist	EEG signal
WDFHO	161	–	1	Linear actuator	Gears	Hand	Joint angle (wrist)
Wearable glove with incorporated compliant mechanical transmission	162	–	2	Pneumatic actuator	Pneumatic	Hand	Manual selection (touch screen)
Wearable mechanism to suppress axial vibration	36	268	3	DC motors	Direct	Wrist	Tremor
WearME Glove	163	500	3	Brushless DC motors	Pulley	Hand and wrist	Joint angle
W-EXOS	164	1900	3	DC motors	Gears	Wrist	Muscle torque and EMG signal
WHOs	165	–	1	Motors	Bar linkage	Hand	Joint angle (wrist)
Wireless distributed FES system	166	45	–	Electrical stimulation	Muscle contraction	Hand	EMG and joint movement
Wireless wearable device	167	–	2	Electrical stimulation	Muscle contraction	Hand and wrist	Joint position and movement (wrist)
Wrist exoskeleton	168	288	2	Linear actuator	Push-pull cable	Wrist	Manual selection
Wrist exoskeleton	169	728	1	DC motors	Gears	Wrist	Mirrored motion
X-Glove	170	–	5	Linear actuator	Cable	Hand	Manual selection

Recordings on the weight of the device were poor in the literature with only 52% (63/121) mentioning weight. Weight spanned from 30g (Layer jamming-based soft Tremor Suppression Glove^34^) to 205kg (Armeo Power II^50^). From the limited reported data, there were indications that the weight of the device on the upper limb was reduced each year on average. The degrees of freedom (DoF) were reported in 62% (84/135) of studies and tended to be low, with many devices actuating one (11%, 13/121) or two DoF (24%, 29/121). Assistive devices which actuated 1 DoF had the lowest weight on average at 285g, followed by 6 DoF at 422g. Devices with higher levels of DoF tended to be designed for the hands: Average hand device DoF was 3.6, whereas wrist devices were 2.8 DoF.

The categories of mechanical transmission described in **Table 2**, were inspired by Bos et al structured overview of dynamic hand orthoses.^17^ However, this study included muscle contraction and supernumerary devices. This improves the inclusivity of unconventional actuation methods; muscle contraction due to electrical stimulation acts as an internally applied active force, and supernumerary devices use indirect mechanical force to attain ADLs. Muscle contraction (26%, 31/121), bar linkage (15%, 18/121) and pneumatic devices (15%, 18/121) were among the most popular mechanical transmission methods across all applications.

To apply the active force, a command signal must be sent to a control unit. This command signal was charted as the “user intent” defined in **Table 2**, results are shown in **Table 3**. The user’s intention to control the device was detected predominantly with Electromyography (EMG) (30%, 36/121) and users’ joint movement (30%, 37/121). The placement of electrodes for EMG varied widely and most EMG intention methods were combined with muscle contraction to actuate the upper limb (61%, 22/36), this is the foundation of Functional Electrical Stimulation (FES).^171^ Other user intention methods include detecting a force applied by the joint typically the fingertips, by manually selecting how and when the actuator moves using a touchscreen or joystick, and EEG systems such as the Emotiv.^65^

### Outcome measures

**Figure 2:**
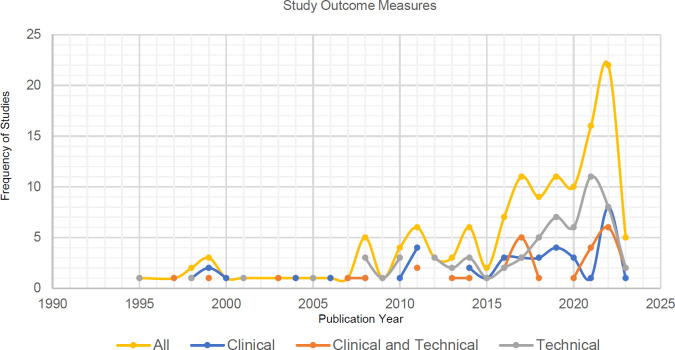
Distribution of studies based on the field of outcome measures.

A total of 226 unique outcome measures were extracted from the 765 tests completed in the data set. From the 226 outcome measures extracted, 100 were considered clinical tools using the WHO-ICF Model of functional outcomes alongside additional validated sources.^10,11,172,173^ Therefore, 126 outcome measures were considered technical or non-clinical. A dip in the number of clinical-based outcome measures used was found in 2020. While testing of devices on patients had decreased, the past 10 years have seen an exponential increase in research publications on upper limb devices seen in **Figure 2**.

**Figure 3:**
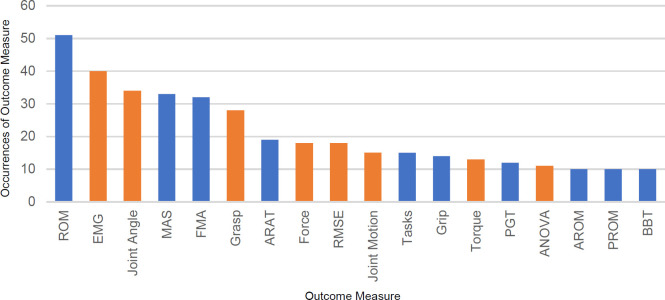
Count of outcome measures. Blue indicates clinical outcomes; orange indicates technical outcomes. The outcome measures in order: ROM (Range of Motion), EMG, Joint angle, MAS (Motor Assessment Scale), FMA (Fugl-Meyer Assessment), Grasp, ARAT (Action Research Arm Test), Force, RMSE (Root Mean Square Error), Joint motion, Tasks, Grip, Torque, PGT (Pinch Grip Test), ANOVA (Analysis of variance), AROM (Active ROM), PROM (Passive ROM), BBT (Box and Blocks Test).

The frequency of outcome measures repeated between studies tended to be low (8%, 18/226). The majority of outcome measures appeared in less than 10 studies (92%, 208/226); **Figure 3** presents the outcome measures most regularly used (outcome measures used in ≥10 studies).

The clinical outcome measures trended towards observational ordinal scales inspecting mobility (MAS, FMA, and BBT) and movement functions (ROM, ARAT and functional tasks). The technical and non-clinical outcome measures were either statistical analysis methods (RMSE, ANOVA and kinematic analysis) or usability tests (EMG, joint angle, and grasp force of device). Patient-reported outcome measures such as the Motor Activity Log (MAL), ABILHAND, Disabilities of the Arm, Shoulder, and Hand Scale (DASH), and quickDASH were often under-utilized with 4% use out of all tests extracted (30/765).

Factors such as introducing variations and modifications in a validation method caused 65% (148/226) of the outcome measurement tools to only be present in the dataset once. Another factor increasing the number of unique outcome measures extracted is the use of condition-specific outcome measures such as the Stroke Impact Scale.^38,110,134,152^ These tailored methods are useful tools to benchmark a person’s functionality within a set population^173–176^ but make validation across different cohorts difficult as it may not be an appropriate outcome measure for all.

### Technology Readiness Levels

TRL 1 (proof of concept studies) and TRL 2 (software prototype studies) were not present due to our inclusion criteria provided in **Table 1**. The distribution of all TRL extracted can be found in **Table 4**, which also includes the definitions used in the data extraction.

**Table 4:** TRL Definitions defined by the HORIZON 2020 - Work Programme 2014-2015 and the scoping review abstraction of the HORIZON 2020 definitions.

TRL	Definition	Scoping Review E[]planation	Count
**1**	Basic principle observed	The idea has been formulated, proof of concept only	0
**2**	Technology concept formulated	A software prototype has been made and tested virtually	0
**3**	Experimental proof of concept	Analytical studies and feasibility studies. The device must be built	17
**4**	Technology validated in the lab	The device has been tested on non-human or one healthy case study for validation.	26
**5**	Technology validated in the relevant environment validation.	The device has been tested on healthy participants	21
**6**	Technology demonstrated in the relevant environment	The device has been tested on a target population in a clinical setting (ISO Standard not complete)	29
**7**	System prototype demonstration in an operational environment	The device has been tested for its intended purpose in an operational environment (outside of the clinic and lab) ISO Standards should be complete	14
**8**	System complete and qualified	The device is ready to be commercialized and has been validated	1
**9**	Actual system is proven in an operational environment	The device is available in the market	27

Overall, TRL 6 (21%, 28/135), TRL 9 (20%, 27/135) and TRL 4 (19%, 26/135) were the most prominent advancement levels. FES (63%, 17/27), EMG (44%, 12/27) and devices made to support people with cardiovascular diseases (74%, 20/27) made up most of the technological advancements of TRL 9. Non-electrical stimulation devices at TRL 9 included the MyoPro,^109–112^ which uses an EMG threshold for control and has been commercialized since 2006, the SEM Glove,^138^ ReoGo-J,^38^ and Armeo Power II.^50^ Of these, SEM Glove, ReoGo-J, and Armeo Power II were the only TRL 9 devices that did not include FES or EMG.

Trends in TRL and demographics were also noticed; as the number of participants increases, the TRL level improves: case studies (1 participant) were an exemption to this trend. High-income countries also conducted studies at higher TRL and there has been a steady development in TRL in device testing over the years.

Devices in category TRL 3 were proof of concept (**Table 4**), therefore these studies use analytical or feasibility methodologies. These methods focus on the validation of the device and include only healthy participants. Of these TRL 3 devices, 47% (8/17) used cable conduit mechanisms, and 29% (5/17) used pneumatic actuation. These devices tended to be designed for supporting the hands (47%, 8/17) and had on average between 2-3 DoF. Various user detection methods were charted, but manual control of the device was quite frequent in both TRL 3 and 9.

## DISCUSSION

This study provides an overview of 135 research papers focused on actuated assistive devices for the hand and wrist. A notable result was the scarcity of rigorous clinical methodologies, with 34% (46/135) of studies involving clinical trials, of which 12 studies conducted RCTs. From these studies, 121 unique devices were analyzed to scope their intended user populations, design features, validation strategies, and TRLs. Most of the devices were designed for individuals with upper limb impairment due to stroke 46% (597/1310), and a significant proportion of devices had low DoF, particularly for wrist devices at an average of 2.8 DoF. Regarding the design, the devices predominantly utilized EMG (30%, 36/121) which tended to be in combination with muscle contraction via electrical stimulation (FES). Along with EMG, user interfaces such as buttons, joysticks, and touch screens were used to detect user intentions. The study categorized a total of 226 unique clinical and technical outcome measures. The validation methods predominantly relied on statistical analyses for technical outcomes, while clinical assessments were often observational. There was a lack of consistency across studies, with many outcome measures used only once (65%, 148/226). Objective or patient-reported outcomes were less frequently employed.

Most of the studies were conducted in high to upper-middle-income economies (90%, 28/31). Although the need for assistive technology in low-income countries is high, there may be a lack of awareness and access to actuated devices, contributing to fewer studies conducted in these economies.^15,16^ Low-income economies must often import medical equipment,^177^ therefore these actuated devices must achieve high TRL to be considered for ordering and prescription. Yet, these devices have not met TRL >6 requirements (76%, 92/121). To fulfil TRL >6, the device must meet the ISO standards, and regulatory requirements (such as CE marking) before distribution in the market or testing in operational environments (**Table 4**). These conditions provide insurance for device quality, safety and efficiency.^178^ A few factors which may contribute to these devices not surpassing TRL 6 include overcoming the dynamic and rapidly developing policies to meet regulatory requirements for testing,^179^ a lack of streamlined clinical tests and validation processes for these devices,^180,181^ and the effects of COVID-19 on reduced face-to-face research.^182–184^

To validate these devices, 226 outcome measurement tools were charted. Classification of validation methods showed that 44% (99/226) of the outcome measurement tools were considered clinical; ROM, MAS and FMA were the most used for clinical trials whereas EMG, joint angles and device grasp force were conducted in technical studies (**Figure 2**). Since many of the devices were designed for stroke rehabilitation (46%, 597/1310), the outcome measures recorded show a strong correlation with existing literature on upper limb outcome measures in stroke recovery.^173^ Patient-reported outcome tests were implemented 4% of the time (30/765). This value is considerably low as these outcomes are invaluable to validate the use of the assistive device.^8,173,185^ Patient-reported outcomes also provide valuable psychometric properties to the evidence base^185^ and are an important part of upper limb assessment. It should be noted that comorbidities were not often reported, and outcome measures were not standardized, therefore inter-comparability of devices and populations was limited.

The lack of inter-comparability was also noticed in the inconsistency in reporting device specifications. DoF and weight of the device were not reported routinely (62% and 52% respectively), with some studies quoting their device as “lightweight” without reference to their objective weight. A slight trend toward reducing the weight of upper limb devices over the years was observed, but there is insufficient statistical evidence to support this claim. Many devices were designed with low (1 or 2) DoF and varied greatly in weight from 33 g to 205 kg. The variation in weight was due to differences in reporting weight, some studies report weight on the upper limb, while others report weight of the full system. The implication of these differing reporting styles makes synthesizing findings difficult for decision-making and provides barriers to further research as the evidence base lacks standardized measures and methods. To improve inter-comparability, frameworks for development can be implemented,^186,187^ alongside robust and systematic testing using a large cohort.^187,188^

In line with the works of Zhu et al, the field of soft wearable robotics has experienced rapid growth^189^ as demonstrated by the increasing number of fluidic transmission actuators identified in the study. These fluidic actuators, which include pneumatic and hydraulic, are typically lighter (averaging 234 g on the arm) and provide multidirectional force due to their flexible design.^190^ Previous studies have predicted the rise of soft robotics,^187^ which may continue to improve for use as an actuated assistive device. In addition to fluidic transmission actuators, supernumerary devices (n = 5) have shown potential for human augmentation.^191^ However, due to their state-of-the-art nature, the availability of real-world applications and longitudinal evidence supporting their effectiveness is limited.^191,192^ As these novel actuators continue to advance, future assistive devices should integrate them to improve weight and multifunctionality.

To detect a user’s intention, EMG (30%, 36/121) and joint movement (30%, 37/121) sensors were regularly implemented. EMG control methods, which include surface electrodes, implanted wires, and probes,^193^ have a long history of use. However, they are not suitable for all individuals with hand and wrist impairment^194^ and may encounter system failures outside of testing settings.^187^ EMG and joint movement sensors are limited by muscle activation threshold requirements, making them inadequate for addressing the full spectrum of people with upper limb impairment. The prescription of these devices would not be appropriate. Consequently, alternative user intention systems were explored including tongue-based interfaces,^71,152^ hands-free voice control,^44^ and foot-based interfaces.^81^ These systems are not limited by upper limb muscle threshold, yet they did not attain TRL >6. Alongside the requirements for attaining TRL >6, design factors may contribute to why these devices are not suitable for operating in a real-world context. Wearable sensing and control technology includes various elements which were not abstracted such as cost, consumption and battery lifespan, these may all affect useability.^187,195^ A systematic analysis of control systems which do not require upper limb muscle activation may be appropriate to validate the use of these underrepresented systems.

### Limitations

The results of a scoping review are often quite broad; a synthesis of the conclusions will require additional resources to be used in policymaking. In addition, scoping reviews rarely include critical appraisal of included studies; therefore, the reliability of findings may be skewed. Despite this, a scoping review addresses the exploratory nature of upper limb devices compared to other methodologies.

In addition, as with many studies, the design of this study is subject to limitations. These concerned the selection of studies, definitions of terms during screening and the exclusion of data charting items. Due to time constraints, this study did not screen all forms of grey literature such as market reports, patents or working papers, and the keyword selection may have excluded appropriate studies. In addition, 118 studies were not retrieved (**Figure 1**) due to restricted access to certain relevant research papers. This limitation arose primarily due to paywalls and institutional access restrictions. This introduced selection bias and may have hindered the scope and number of devices investigated with higher technological readiness levels. During the screening process, the reviewers ultimately agreed on a consensus with 86.9% accuracy, but the definition of portable was defined as easily moveable by healthy users. This meant results on the weight of the device had large variability. This limitation was somewhat mitigated by recording the device's weight on the arm, although some studies only reported the total weight of the device. This study did not chart how the device interacts with the user’s joint-segment, such as enabling voluntary hand-opening or supporting wrist flexion. This data charting item would have provided more context for the device's functions.

## CONCLUSION

Active, actuated assistive devices offer promising solutions to improve functionality and quality of life for individuals with hand impairments. This study reviews 135 studies covering 121 devices, providing insights into actuated devices for hand and wrist support in ADLs. Innovation in actuation systems and control methods is evident, yet many devices have not advanced beyond TRL 7, highlighting the gap between research and market-ready products. EMG and FES systems dominate the field but may not be suitable for users with limited muscle activation, showing the need for alternative approaches such as tongue interfaces and voice control systems.

Key barriers to prescription included insufficient real-world evidence, concentration of development in high- and middle-income countries, lack of standardized reporting, and the absence of accepted clinical validation processes. To overcome these challenges, it is essential to establish standards for device design, testing, and reporting (e.g., weight, degrees of freedom), develop comprehensive outcome measures combining objective methods with patient-reported experiences, and improve the accessibility of devices in low-income countries.

The field of hand and wrist exoskeletons shows increased popularity in the innovation of control systems and actuators. Addressing these challenges and implementing standardized frameworks will help improve the prescription of these devices. As technology advances, tailored solutions for individuals with varying levels of hand functionality are becoming increasingly feasible, offering significant benefits to those with upper limb impairments. Overall, there is promise and growth in the field of hand and wrist exoskeletons.

## DECLARATION OF CONFLICTING INTERESTS

The authors declare no conflict of interest.

## AUTHORS’ CONTRIBUTION

- **Angel Galbert:** Study conception and design, data collection, analysis and interpretation of results, draft manuscript preparation, and manuscript revision.
- **Arjan Buis:** Supervision, study conception and design, and manuscript revision.

Both authors have read and approved the final version of the manuscript.

## SOURCES OF SUPPORT

ESPRC doctoral training grant (EP/S02249X/).

## List of Abbreviations

ADL: Activities of Daily Living
AHA: Assisting hand assessment
AHP: Allied Health Professional
AMEA: Absolute Mean Error Analysis
ANCOVA: Analysis of Covariance
ANOVA: Analysis of Variance
AOU: Amount of Use
ARAT: Action Research Arm Test
ARI: Active Resistance Index
AROM: Active Range of Motion
ASIA: American Spinal Injury Association (ASIA) Impairmen Scale
BBT: Box and Block Test
BI: Barthel Index
BMRC: British Medical Research Council Scale
CGI: Clinical Global Impression
Chedocke: Chedocke McMaster Hand Portion
CMC: Coefficient of Multiple Correlation
CMSA: Chedocke-McMaster Stroke Assessment
COPM: Canadian Occupational Performance Measure
CTS: Carpal Tunnel Syndrome
CUE-T: Capabilities of upper extremity test
CVA: Cerebral Vascular Accident
DMD: Duchenne Muscular Dystrophy
DOF: Degree of Freedom
Donn/Doff: Putting on and Removing Task
D-QUEST: Dutch-Quebec User Evaluation of Satisfaction with Assistive Technology
DTM: Dart throwing motion
DTSaM: Dynamic Traction Splint by Artificial Muscle
EEG: Electroencephalogram
EMG: Electromyography
FAT: The Frenchay Arm Test
FEA: Finite Element Analysis
FES: Functional Electrical Stimulation
FIM: Function Independence Measurement
FMA: Fugl-Meyer Assessment
fMRI: Functional magnetic resonance imaging
GAIN: Global Appraisal of Individual Needs
GRASSP: Graded Redefined Assessment of Strength, Sensibility and Prehension
GRT: Grasp and Release test
IBEP: Integral Value of a Bioelectric Potential
IMU: Inertial measurement units
IOTA: Isolated orthosis for thumb actuation
IRQ: Interquartile range
JTHFT: Jebson Taylor Hand Function Test
KINARM: Kinesiological Instrument for Normal and Altered Reaching Movement
LGMD: Limb Girdle Muscular Dystrophies
MAL: Motor Activity Log
MANOVA: multivariate analysis of variance
MAPR: Multi-Attribute Preference Response
mARAT: Modified Action Research Arm Test
MARP: Mean arrest period ratio
MAS: Modified Ashworth Score
MAV: Mean Absolute Value
MDC: Minimal detectable change
MES: Mean Error Squared
MMSE: Mini-Mental State Examination
MMT: Manual Muscle Testing
Movement ABC: Movement Assessment Battery for Children
MPF: Mean Power Frequency
mPPT: modified Purdue Pegboard Test
MVC: Maximum Voluntary Contraction
NASA-TLX: The NASA Task Load Index
NHPT: Nine Hole Peg Test
NIHSS: National Institute of Health Stroke Scale
NSA: Nottingham Sensory Assessment
PCGI-I: Patient Clinical Impressions-Improvements
PIADS: Psychosocial Impact of Assistive Devices Scale
PRISMA-ScR: The Preferred Reporting Items for Systematic Reviews and Meta-Analysis Extension for Scoping Reviews
PROM: Passion range of motion
PRS: Pain Assessment Rating Scale
PUL: Performance of the Upper Limb scale
QIF-SF: Quadriplegia Index of Function-Short Form
QoL: Quality of Life
QOM: Quality of movement scale
QUEST: Quebec User Evaluation of Satisfaction with Assistive Technology
QuickDAS H: Quick Disabilities of the Arm, Shoulder, and Hand Questionnaire
RCT: Randomized Control Trial
RMA: Rivemead Motor Assessment
RMSD: Root means square difference
RMSE: Root means square error
RMSED: Root Mean Standard Error of Deviation
ROM: Range of motion
RTLX: Raw NASA-Task Load Index
SAL: Spectral arc length
SCIM-SR: Spinal Cord Independence Measure–Self-Report
sEMG: Surface Electromyography
SEPs: Somatosensory evoked potentials
SIAS: Stroke Impairment Assessment Set
SIS: Stroke Impact Scale
SUS: System Usability Scale
SWMT: Semmes-Weinstein monofilament test
TAM: Total Active Motion
TBI: Traumatic Brain Injury
TLT: Thumb localizing test
TRI-HFT: Toronto Rehabilitation Institute Hand Function Test
TRL: Technology Readiness Level
UDQ: Use of Device Questionnaire
USE: the Usefulness-Satisfaction-and-Ease-of-use-Questionnaire
VAS: Visual Analog Pain Assessment Scale
WHO-ICF: World Health Organisation - The International Classification of Functioning, Disability and Health
Wilcoxon Test: The Wilcoxon signed-rank test
WMFT: Wolf Motor Function Test
